# Research status and hotspots of social frailty in older adults: a bibliometric analysis from 2003 to 2022

**DOI:** 10.3389/fnagi.2024.1409155

**Published:** 2024-06-05

**Authors:** Hengxu Wang, Xi Chen, MingXiang Zheng, Ying Wu, Lihua Liu

**Affiliations:** ^1^School of Medicine, Hunan Normal University, Changsha, China; ^2^The Second Xiangya Hospital, Central South University, Changsha, China; ^3^Clinical Research Center for Reproduction and Genetics in Hunan Province, Reproductive & Genetic Hospital of CITIC-Xiangya, Changsha, China; ^4^NHC Key Laboratory of Human Stem Cell and Reproductive Engineering, School of Basic Medical Sciences, Central South University, Changsha, China

**Keywords:** older adults, social frailty, research trends, research hotspots, visual analytics, bibliometric

## Abstract

**Background:**

Social Frailty is a significant public health concern affecting the elderly, particularly with the global population aging rapidly. Older adults with social frailty are at significantly higher risk of adverse outcomes such as disability, cognitive impairment, depression, and even death. In recent years, there have been more and more studies on social frailty, but no bibliometrics has been used to analyze and understand the general situation in this field. Therefore, by using CiteSpace, VOSviewer, and Bilioshiny software programs, this study aims to analyze the general situation of the research on social frailties of the older adults and determine the research trends and hot spots.

**Methods:**

A bibliometric analysis was conducted by searching relevant literature on the social frailty of the older adults from 2003 to 2022 in the Web of Science core database, using visualization software to map publication volume, country and author cooperation networks, keyword co-occurrences, and word emergence.

**Results:**

We analyzed 415 articles from 2003 to 2022. Brazil has the highest number of articles in the field of social frailty of the older adults, and the United States has the highest number of cooperative publications. Andrew MK, from Canada, is the most published and co-cited author, with primary research interests in geriatric assessment, epidemiology, and public health. “Social Vulnerability,” “Health,” “Frailty,” “Mortality,” and “Older Adult” are among the research hotspots in this field. “Dementia,” “Alzheimer’s disease,” “Population,” and “Covid-19” are emerging research trends in social frailty among the older adults.

**Conclusion:**

This scientometric study maps the research hotspots and trends for the past 20 years in social frailty among the older adults. Our findings will enable researchers to better understand trends in this field and find suitable directions and partners for future research.

## 1 Introduction

Frailty is a significant public health concern affecting the elderly, particularly with the global population aging rapidly. It was first proposed at the American Federal Conference on Aging in 1987. Fried et al. (2001) define frailty as a clinical syndrome that occurs when age-related factors lead to a decline in physiological reserve, causing an increase in the body’s vulnerability and a decline in its ability to cope with pressure. The types of frailty mainly include physical frailty, cognitive frailty, and social frailty. Although research on frailty has mostly focused on physical decline, studies have shown that social frailty is an important dimension that is closely related to overall frailty. In fact, social frailty can precede and contribute to overall frailty (Andrew et al., 2008). Despite this, research on social frailty has not received as much attention as physical and cognitive frailty. Past research has demonstrated that social frailty is strongly associated with various negative health outcomes in the elderly, such as depression, anxiety, obesity, cardiovascular disease, and increased hospitalization, disability, mortality, and cognitive impairment rates (Makizako et al., 2018; Park et al., 2019; Tsutsumimoto et al., 2019). In 2017, Bunt introduced the comprehensive concept of social frailty based on the social demand of the social productive function theory, wherein an individual continuously loses one or more important resources to meet basic social needs, which results in a lack of social behavior, activities, and self-management ability (Bunt et al., 2017). Studies have shown that older adults with social frailty are at significantly higher risk of adverse outcomes such as disability, cognitive impairment, depression, and even death than other older adults (Teo et al., 2017).

A meta-analysis revealed a high prevalence of social frailty among the older adults, with an aggregate prevalence of 47.3% among hospitalized older adults individuals and 18.8% among those living in the community (Zhang et al., 2023). Notably, social frailty may occur earlier than physical frailty (Makizako et al., 2018), but its significance is often overlooked. Given the increasingly severe aging trend in China, where over 50% of older adults individuals are empty-nesters, it is estimated that by 2030, empty-nesters will account for 90% of the total number of older adults (Su et al., 2020). The lack of companionship from family and friends can reduce the degree of family care (Shamsikhani et al., 2023) and social participation (Su et al., 2020), potentially leading to social frailty among the older adults. Therefore, further exploration and prediction of social frailty in the older adults are crucial.

Bibliometrics, as a novel scientific measurement technique, offers a comprehensive qualitative and quantitative approach to studying publications. It focuses on the quantitative attributes of literature, conducting in-depth analyses to identify multidimensional features such as countries, institutions, journals, authors, and keyword distributions within specific fields over defined periods (Zhu et al., 2023). This systematic approach enables researchers to discern prevalent topics within a research domain, accurately depict academic trends, and anticipate future research frontiers (Chen et al., 2012). Bibliometrics not only provides robust data support for current research but also establishes a firm academic foundation for shaping future research directions and strategies. While traditional literature reviews, systematic reviews, and main path analyses can offer quantitative insights, bibliometrics uniquely enable simultaneous analysis of author, institution, country, and journal contributions and collaborations within the academic realm, facilitating precise assessments of knowledge bases and research focal points (Chen and Song, 2019). In recent years, there have been more and more studies on social frailty, but no bibliometrics has been used to analyze and understand the general situation in this field. Therefore, by using CiteSpace, VOSviewer, and Bilioshiny software programs, this study aims to analyze the general situation of the research on social frailties of the older adults and determine the research trends and hot spots in the past two decades from 2003 to 2022, to provide references for researchers to understand hot spots in related fields and seek cooperation.

## 2 Method

### 2.1 Design

This study used CiteSpace, VOSviewer and Biblioshiny software to conduct data analysis, and interprets the literature data on social frailty among the older adults from 2003 to 2022 by analyzing various aspects, such as the number of publications, national cooperation network, core authors, co-cited literature, keyword co-occurrence, and keyword emergence. By examining these dimensions of the literature, the study aims to identify the research hotspots and trends in this field over the past two decades.

### 2.2 Sample

**Figure 1:**
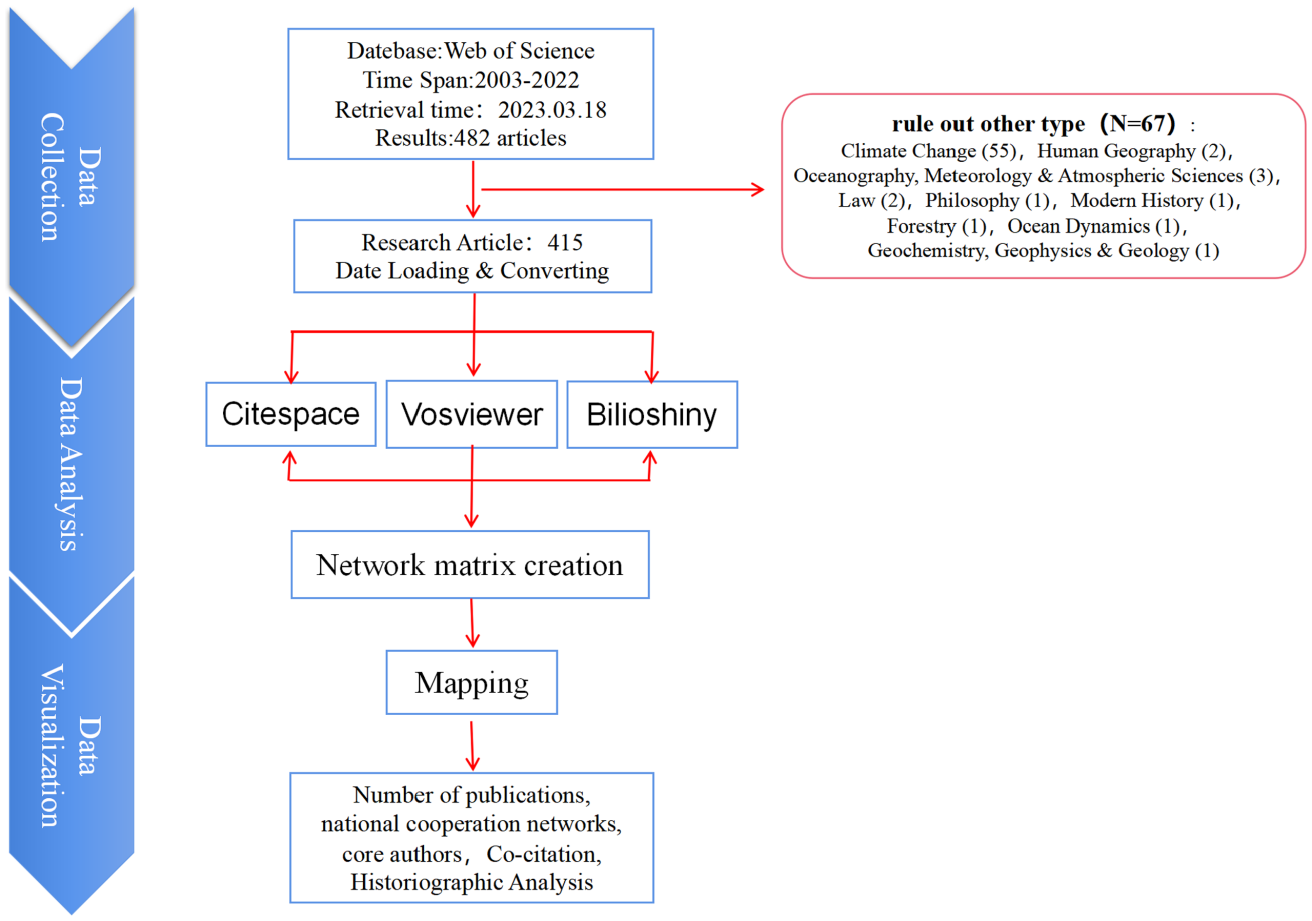
Literature screening and data analysis processes.

A total of 482 articles were retrieved under the specified search conditions. However, 67 non-medical articles that did not fall under the subject classification were excluded (Figure 1). The outcome of this literature search resulted in the derivation of 415 publications.

### 2.3 Data collection

In this study, the Web of Science™ core collection database was utilized as the primary data source to conduct a literature search related to social frailty. The search strategy used was TS = (“aged” OR “elderly” OR “older people” OR “older adult∗” OR “Aged, 80 and over” OR “Frail Elderly”) AND TS = (“social frailty” OR “social vulnerability” OR “social frail∗” OR “Social vulnerabilities”). The search was conducted within the timeframe of January 1st, 2003 to January 1st, 2023, and the retrieval of data was carried out on March 17, 2023. The retrieved literature was exported in plain text format, where the entirety of the text content, including referenced citations, was recorded and saved in the input folder with the file name download_*.txt.

Inclusion criteria: ① Published journal literature on social frailties in the older adults; ② The language is English. Exclusion criteria: ① non-academic documents such as conferences, news, and reports; ② Repeated publication of literature.

### 2.4 Data analysis

In this study, a range of software tools was employed for the analysis of literature data on the social frailty of the elderly from 2003 to 2022. Specifically, Excel was used to create charts for the number of published documents and journal data retrieved. Web of Science was utilized for obtaining the journal impact factor and H index. Subsequently, CiteSpace, VOSviewer, and Biblioshiny were applied to analyze the data. This section mainly describes in the CiteSpace software, settings were adjusted for Time Slicing (2003–2022) and #Years Per Slice (1 year). The Node Types are offered different options such as Author, Country, Institution, and Keywords. The Selection Criteria was set to Top *N* = 50, and Pruning was chosen to include “Pathfinder” and “Pruning the sliced networks.” After these Settings were established, the software was run to obtain the visual map.

### 2.5 Validity, reliability and rigidity

The data used in this study was sourced from high-quality journal publications. To ensure data accuracy and reduce duplication, two researchers carefully reviewed all articles retrieved for the study. Additionally, these researchers reviewed the titles, abstracts, and full texts of all selected literature, following strict inclusion and exclusion criteria aimed at excluding articles that were not relevant to our research topic.

## 3 Results

### 3.1 Number of publications

**Figure 2:**
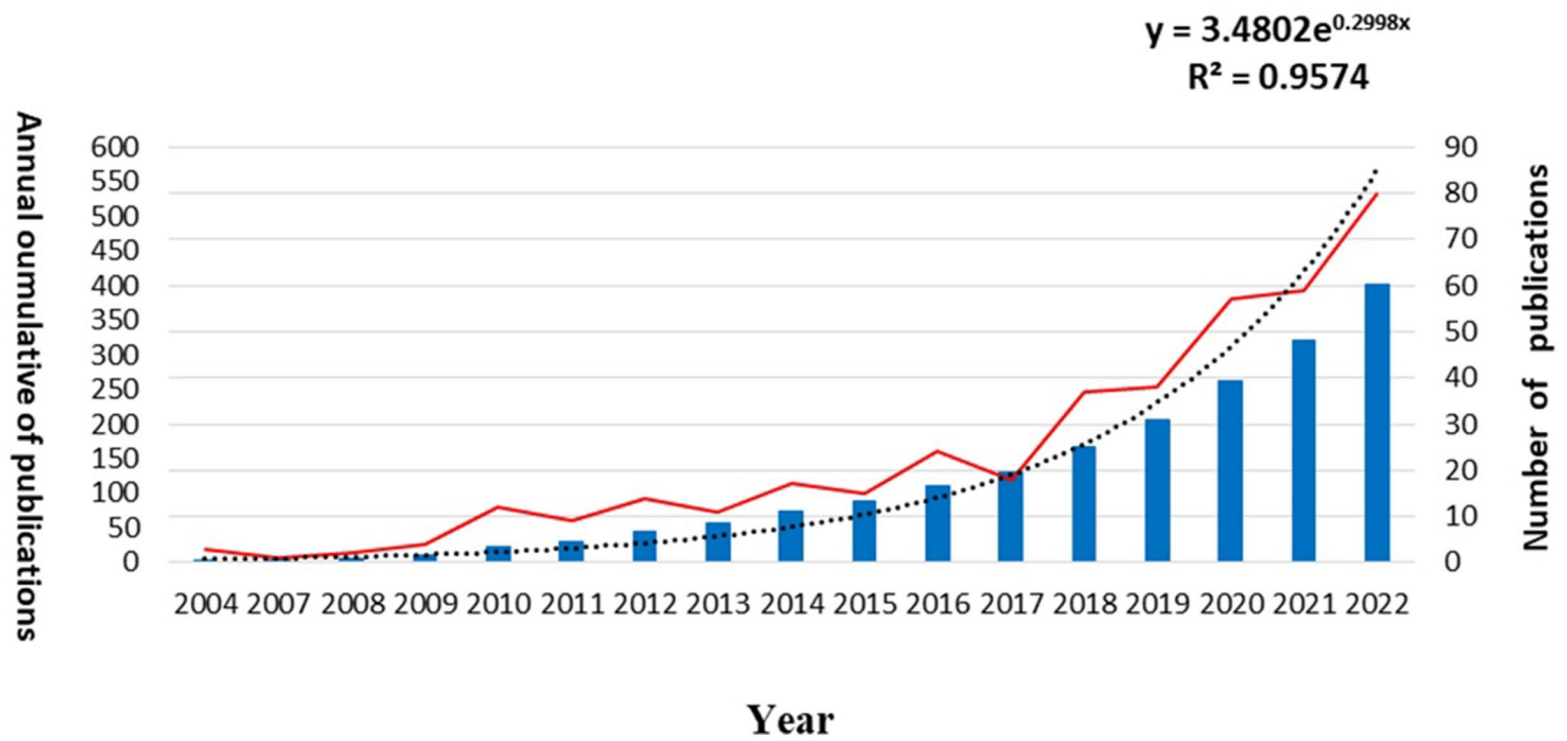
Number of articles published annually in the field of social frailty.

The quantity of literature is a significant indicator for measuring the developmental status of a research field and can provide insight into its future trends (Li and Song, 2022). As illustrated in Figure 2, the trend of the number of publications in international studies on the social decline of the elderly has fluctuated in recent years, but overall, there has been an upward trend. Based on the observed growth pattern, a regression model with a high degree of fitting (*R*^2^ > 0.7) is obtained using the growth equation *y* = 3.4802e^0.2999x^, *R*^2^ = 0.9574. Particularly, the analysis reveals that the period from 2017 to 2022 shows the most significant growth in the number of published papers, suggesting that interest in the social frailty of the elderly has been increasing in recent years.

### 3.2 Cooperation map of country

**Figure 3:**
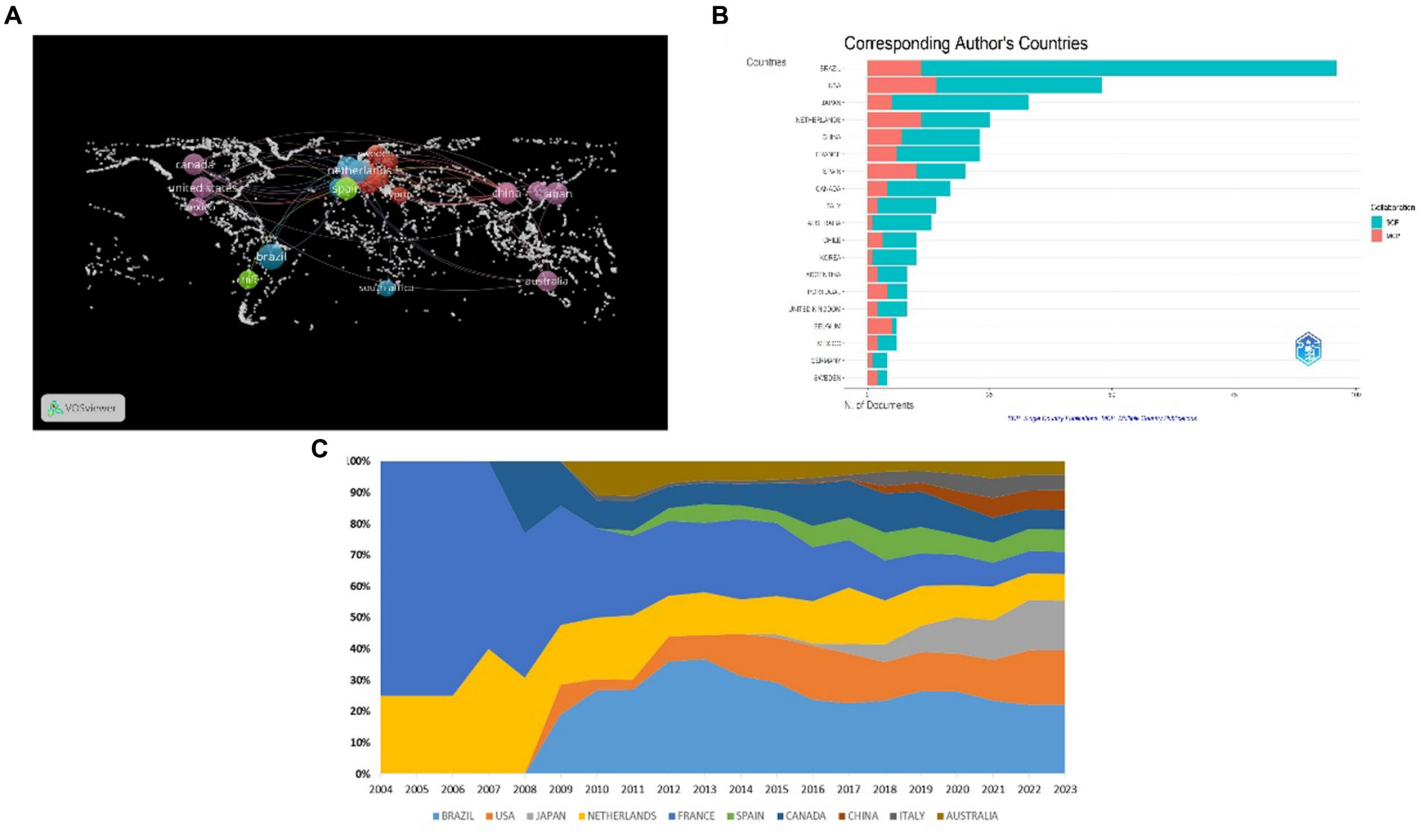
Analysis of national publications. **(A)** Collaboration WorldMap. **(B)** Corresponding author’s countries. **(C)** the area chart of the proportion of national publications.

In this study, we used VOSviewer and Biblioshiny software to create a geographical map of countries and a map of their collaborative publications. The nodes in Figure 3A represent each respective country, with the node size indicating the number of documents issued by the country. Wired connections between nodes indicate countries that collaborate, with more countries that engage in collaboration resulting in more connections. Additionally, the mediation centrality of a node indicates the strength of association and collaboration (Freeman, 1977). Based on the provided data, the top five countries in terms of the number of published articles are Brazil (96), the United States (48), Japan (33), the Netherlands (25), and China (23). On the other hand, the top five countries in terms of mediation centrality, are Brazil (0.62), followed by the United States and the Netherlands both tied with a score of 0.34, China (0.24), and Spain (0.22). In Figure 3B, “single country publications” (SCP) represent the volume of individual country publications; “Multiple Country Publications” (MCP) indicates that many countries have cooperated in publishing articles, of which the United States has the largest number of independent publications in the field of Social frailty, ranking second in total publications; Brazil has the largest number of cooperative publications and the total number of publications, which shows that Brazil and the United States not only have a high number of publications but also pay attention to cooperation, and are in a leading position in the field of Social frailty. Figure 3C shows the area chart of the proportion of national publications, and the results of the analysis are consistent that Brazil and the United States are generally dominant, especially in the early stage of social frailty research (before 2008), and in 2008, French scholars began to study social frailty, and continued to maintain research enthusiasm in the middle and late stages, and the proportion gradually caught up with Brazil.

### 3.3 Author analysis

Co-citation author analysis means that the literature of two authors is cited by the third author at the same time (Zhang et al., 2022), and through the analysis of the authors with the highest number of publications and co-citation frequency, it can intuitively reflect the research strength of the authors and the research hotspots in the field of social weakness. Data analysis showed that there were 1943 authors in the field of social frailty of the older adults, of which 1,697 (87.3%) published one article; 160 (8.2%) published two papers; 33 (1.7%) published three papers; Twenty-five (1.3%) published four or more papers. Authors who have published multiple papers are considered “core” authors. The analysis of the top 10 authors in the field of social frailty found that Andrew MK (Dalhousie Univ, Div Geriatr Med, Halifax, NS, Canada) has the highest number of publications and H-index. Generally, the higher the H-index, the greater the author’s influence in the field, as the H-index accurately and objectively evaluates scholars’ academic influence based on the number of publications and citations (Esposito, 2010). It can also evaluate other academic entities such as countries, research institutions, and academic teams. As the H-index does not factor in the academic age of the author, which refers to the time since the author published their first paper, the *M*-index = (*H*-index/academic age) has been calculated. Additionally, the g-index was used to assess the authors’ most cited papers.

**Figure 4:**
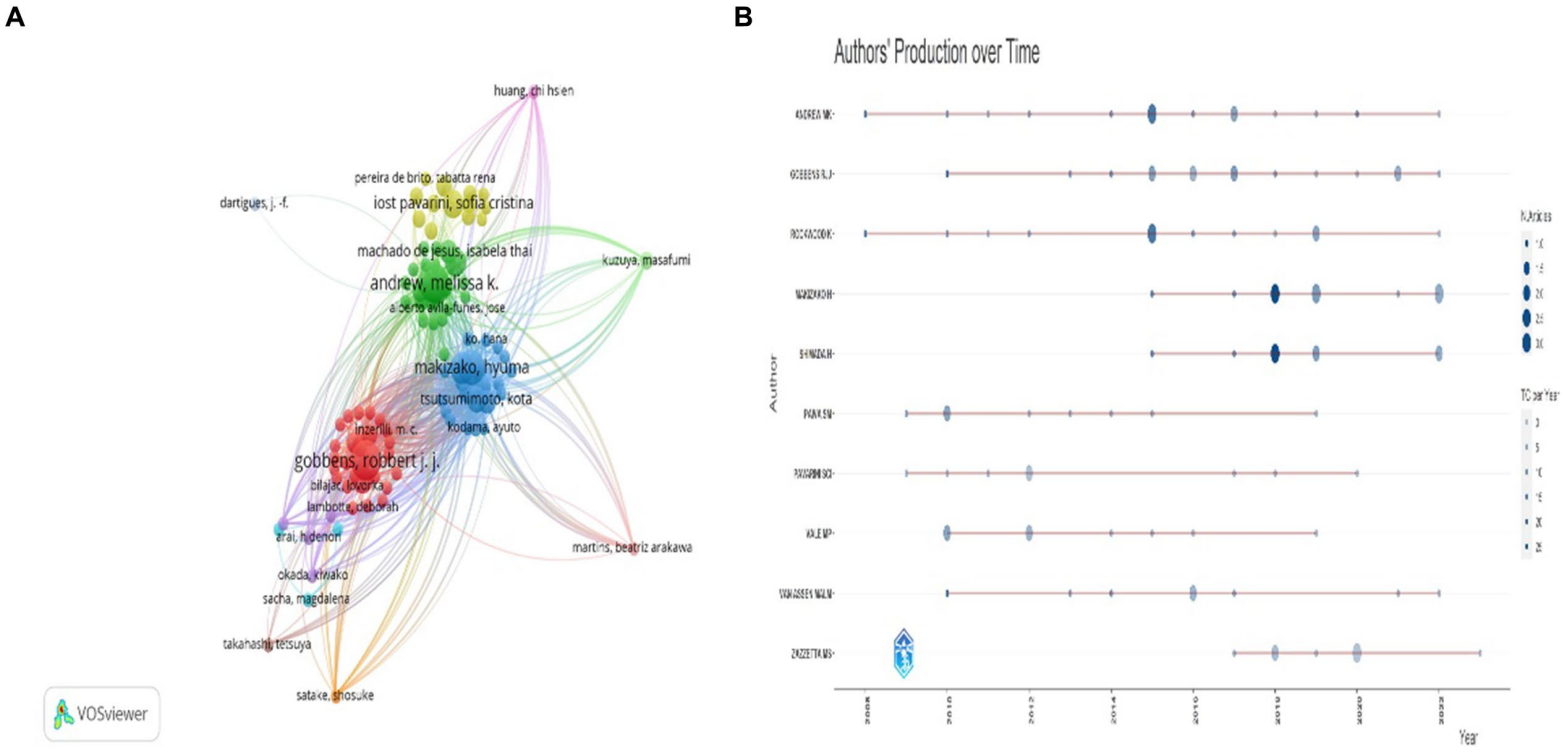
Analysis of the cited authors. **(A)** Co-cited author relationship network map, where nodes of different colors represent the group of authors who form cooperation. **(B)** Authors’ Production Over Time, the node size represents the number of documents, and the color shade represents the average number of citations per year.

Table 1 The top five most cited authors in the field of social frailty were Andrew MK (768), Rockwood K (660), Gobbens RJJ (609), Van Assen MALM (455), and Makizako H (386), indicating that Andrew MK has a high level of influence in both publication volume and co-citations. Figure 4A displays the author’s collaboration network, which can be approximately classified into four different color-coded cooperative groups, namely Andrew MK, Machado De Jesus, IT, etc. (green), Makizako H, Tsutsumimoto K, etc. (blue), Gobbens RJJ, Inzerilli MC, etc. (red), and Iost Pavarini SC, Pereira De Brito TR, etc. (yellow). Furthermore, Figure 4B illustrates the average annual number of publications and contributions of influential authors in the field of social frailty. The figure reveals ANDREW MK as the author with the highest average citation per article, and they have maintained a high scientific impact in the area of social frailty from 2008 to 2022.

**Table 1:** Top 10 authors in the field of social frailty of older adults.

Rank	Author	*H*-index	*G*-index	*M*-index	Publications	Total citation	PY_start
1	Andrew MK	13	15	0.81	15	768	2008
2	Rockwood K	12	13	0.75	13	660	2008
3	Gobbens RJJ	10	15	0.71	15	609	2010
4	Makizako H	7	12	0.78	12	386	2015
5	Paiva SM	7	8	0.47	8	264	2009
6	Shimada H	7	9	0.78	9	372	2015
7	Vale MP	7	8	0.5	8	209	2010
8	Van Assen MALM	7	8	0.5	8	455	2010
9	Pavarini SCI	6	8	0.4	8	67	2009
10	Pordeus IA	6	6	0.4	6	234	2009

### 3.4 Journal publication volume and co-citation analysis

**Figure 5:**
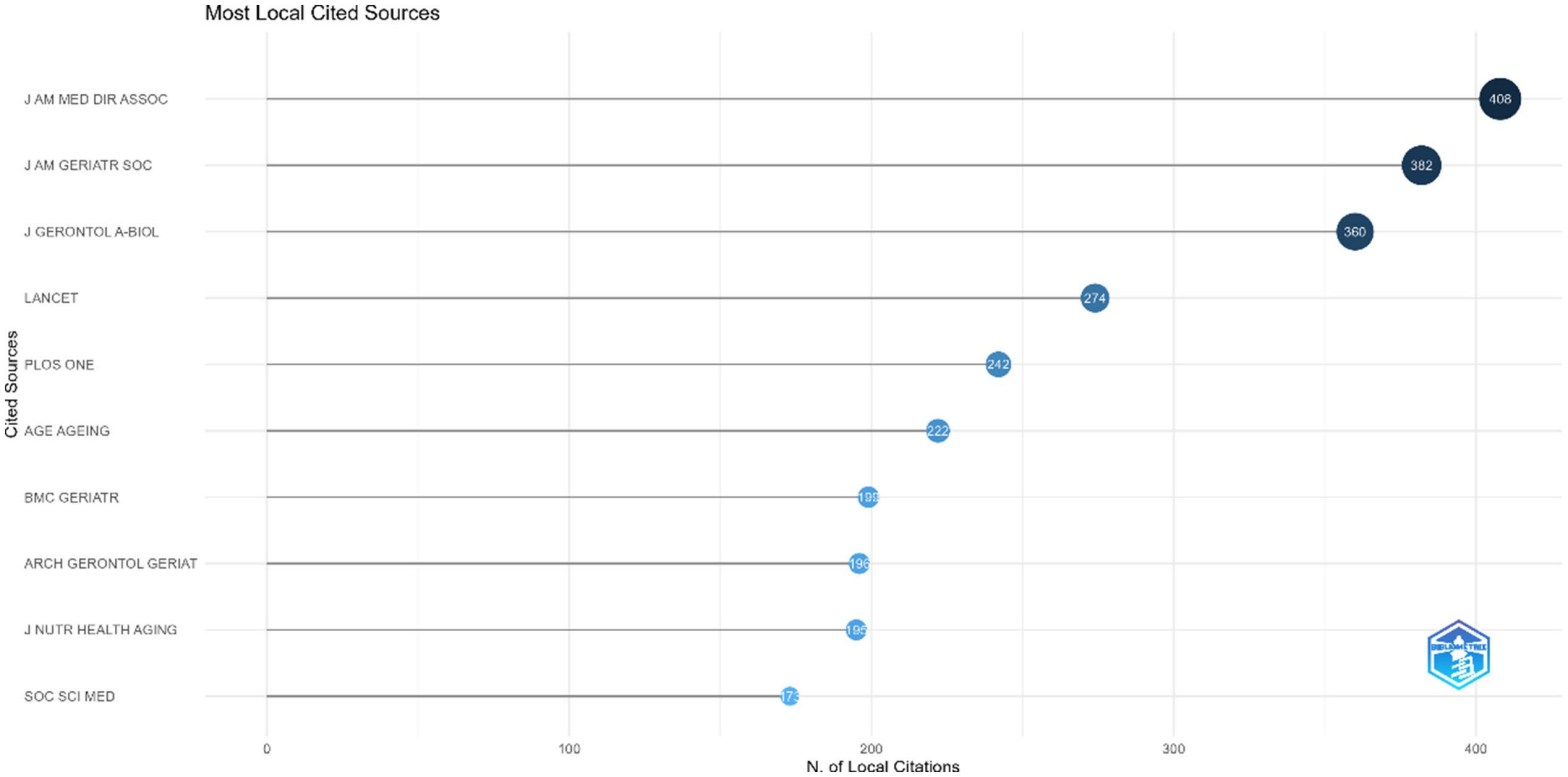
Top 10 journals by total citation.

Table 2 displays the top 10 journals with the most published publications in the field of social frailty. *The international journal of environmental research and public health* ranked first. In Figure 5, the top three most cited journals were *J Am Med Dir Assoc* (408 citations), *J Am Geriatr Soc* (382 citations), and *J Gerontol A-Biol* (360 citations). Journals with more citations suggest that they contain higher-quality articles with wider applicability.

**Table 2:** Top 10 journals with publications related to social frailty.

Rank	Total publications	Year publication	Journal	Publications	Country	H-Index	Impact factor
1	59,389	2004	INT J ENVIRON HEAL R	19	Switzerland	113	4.614
2	7,854	1996	CIENCIA AND SAUDE COLETIVA	14	Brazil	78	1.917
3	4,845	1982	ARCH GERONTOL GERIAT	12	Netherlands	89	4.163
4	4,575	2001	BMC GERIATRICS	12	United Kingdom	89	4.07
5	5,025	2000	J AM MED DIR ASSOC	10	United States	114	7.802
6	2,920	1997	J NUTR HEALTH AGING	7	France	95	5.285
7	281,518	2006	PLOS ONE	7	United States	332	3.752
8	3,515	1967	REV ESC ENFERM USP	5	Brazil	42	1.123
9	2,944	2001	GERIATR GERONTOL INT	5	Serbia	71	3.387
10	10,202	1972	AGE AND AGEING	4	United Kingdom	143	12.782

### 3.5 Analysis of co-cited literature

the number of citations is often used as a measure to determine the quality and impact of scientific literature on the scientific community. It can reflect the attention and influence of a publication, indicating how frequently and widely other researchers reference it in their own work. Higher citation counts suggest that a publication is more impactful and valuable to the research community. Table 3 provides a list of the top 10 cited references in the field of social frailty, which include reviews, cross-sectional surveys, longitudinal studies, and systematic reviews. The main research content covers the definition of social frailty, theoretical frameworks, and influencing factors. The most cited article was a review published by Robertson DA et al. in 2013, with a total of 421 citations and an annual average of 38.27.

**Table 3:** Top 10 citation references.

Year	Author	Article Title	DOI	Total Citations	citations per year
2013	Robertson DA	Frailty and cognitive impairment--a review of the evidence and causal mechanisms	10.1016/j.arr.2013.06.004	421	38.27
2010	Gobbens RJJ	Determinants of frailty	10.1016/j.jamda.2009.11.008	225	16.07
2008	Andrew MK	Social vulnerability, frailty and mortality in elderly people	10.1371/journal.pone.0002232	214	13.38
2017	Bunt S	Social frailty in older adults: a scoping review	10.1007/s10433-017-0414-7	161	23.00
2018	Makizako H	Social Frailty Leads to the Development of Physical Frailty among Physically Non-Frail Adults: A Four-Year Follow-Up Longitudinal Cohort Study	10.3390/ijerph15030490	101	16.83
2015	Makizako H	Social Frailty in Community-Dwelling Older Adults as a Risk Factor for Disability	10.1016/j.jamda.2015.08.023	98	10.89
2014	Gobbens RJJ	The prediction of quality of life by physical, psychological and social components of frailty in community-dwelling older people	10.1007/s11136-014-0672-1	86	8.60
2013	Garre-Olmo J	Prevalence of frailty phenotypes and risk of mortality in a community-dwelling elderly cohort	10.1093/ageing/afs047	80	7.27
2014	Andrew MK	Social vulnerability from a social ecology perspective: a cohort study of older adults from the National Population Health Survey of Canada	10.1186/1471-2318-14-90	78	7.80
2010	Andrew MK	Social vulnerability predicts cognitive decline in a prospective cohort of older Canadians	10.1016/j.jalz.2009.11.001	78	5.57

**Figure 6:**
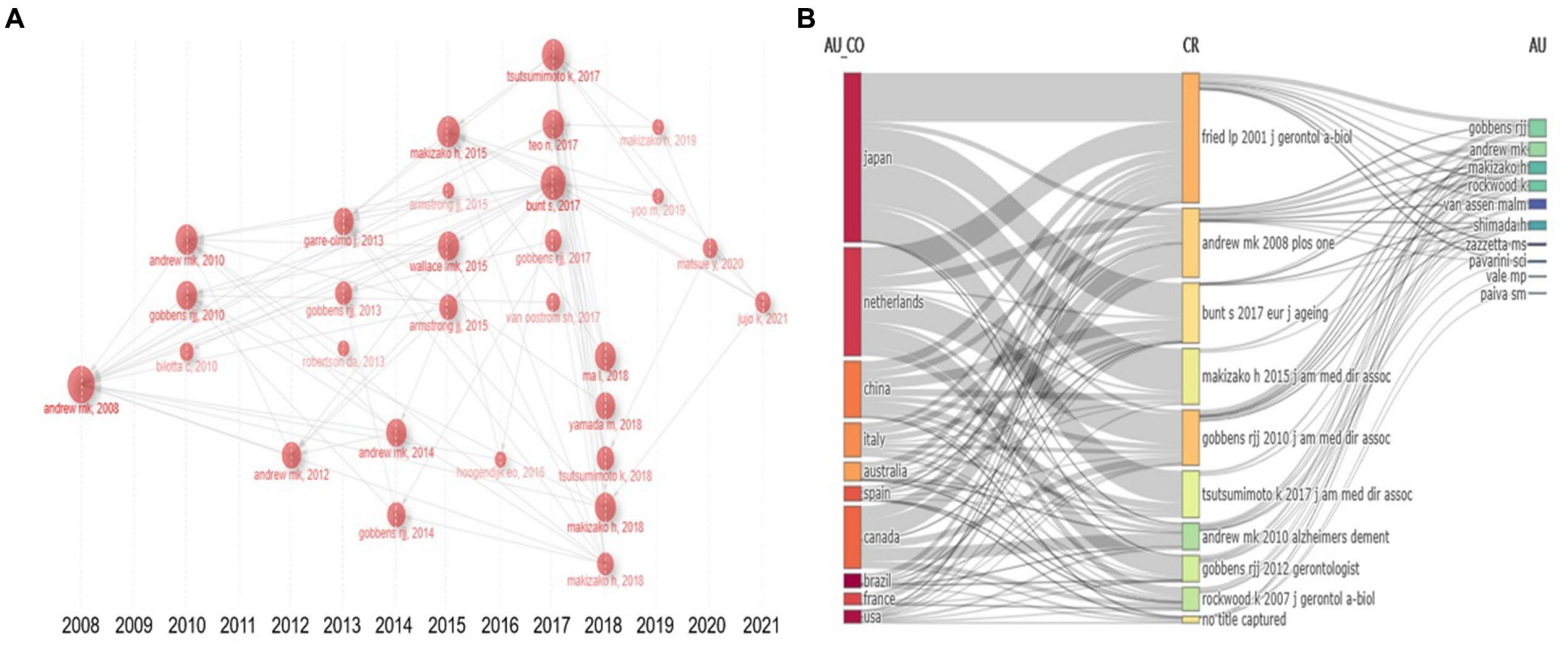
Analysis of co-cited literature**. (A)** The top 30 reference relationship networks by year are arranged in chronological order, with node size indicating the number of citations and arrows pointing to cited documents. **(B)** Three-field plot, the left field is the author country or institution, the middle field is the cited document, and the right field is the author.

By using the Biblioshiny software and selecting “Historiograph” in the “Intellectual Structure” option for analysis, Figure 6A displays node size and label color related to the number of citations, while the lines between documents indicate citation relationships, and arrows point to the cited documents. By analyzing the relationship of references among 30 nodes, the historical process of social frailty research can be understood. Results indicate that ANDREW MK’s 2008 article “Social vulnerability, frailty, and mortality in elderly people” was the most cited social frailty literature, with a total of 52 citations. Regarding years, 2017 (5 articles) was the most cited among literature on the same topics. This suggests that the research on social frailty has continued to increase in popularity and depth. Another noteworthy article is the second most cited article by Bunt S et al. in 2017. They first proposed the comprehensive concept of social frailty based on the concept of social needs in Social Production Function Theory and constructed a social frailty integration model that defined the concept of social frailty comprehensively from a social level, providing a complete concept of social frailty. Therefore, the citation frequency ranks high. In Figure 6B, the relationship between countries, authors, and citations in the field of social frailty is illustrated. The connections between the three regions represent the relationships between each other, and the line width indicates the frequency.

### 3.6 Keyword co-occurrence analysis

**Figure 7:**
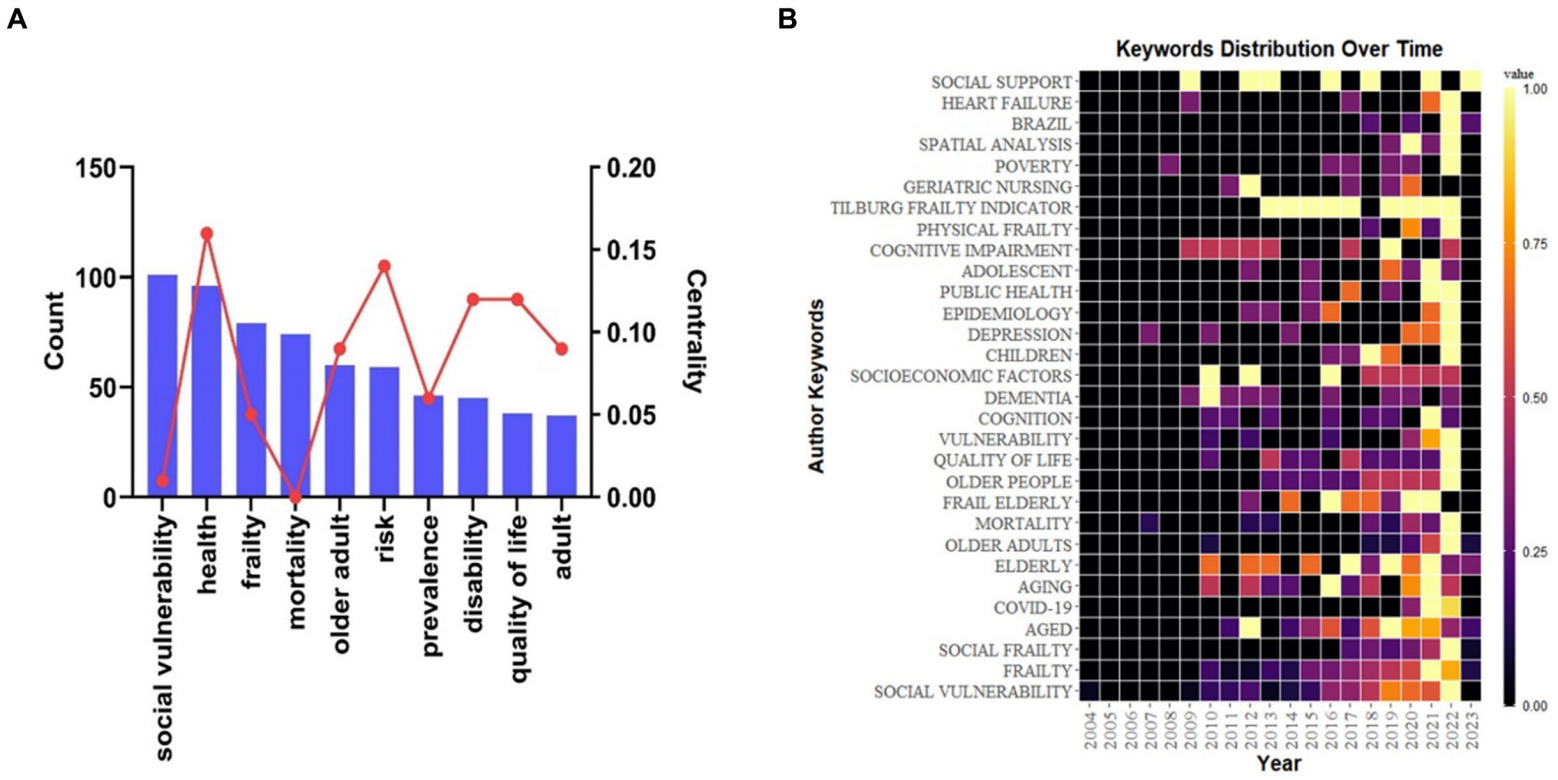
High-frequency keywords and heat maps. **(A)** High-frequency keywords, the top 10 keywords in the field of social weakness, the bar chart is the frequency of the keywords, and the line chart shows the centrality of the keywords. **(B)** Keywords distribution over time, the closer the square color block color is to yellow, the more popular the keywords.

**Figure 8:**
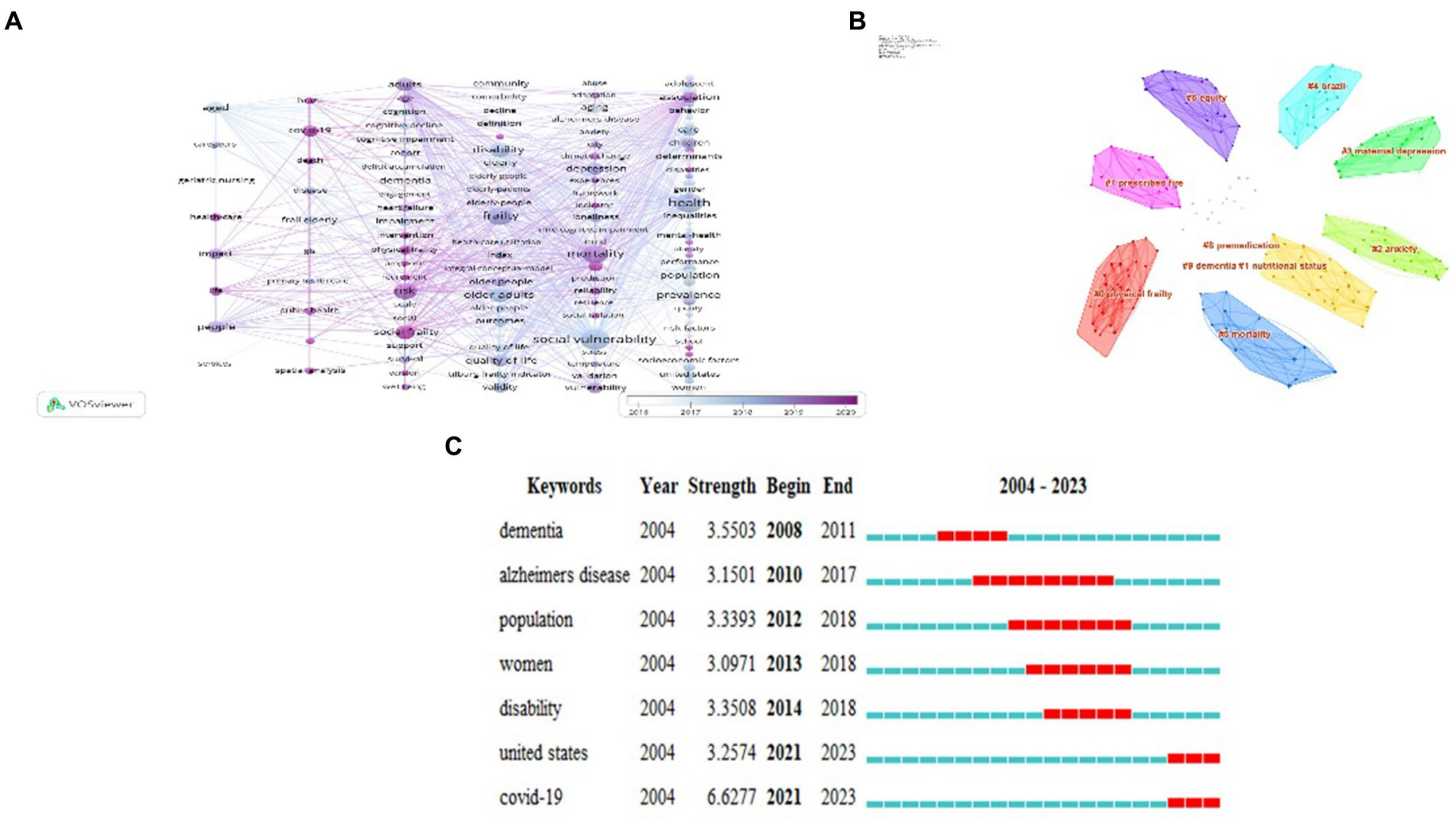
Keyword co-occurrence, clustering and strongest citation bursts analysis. **(A)** social frailty keywords co-appear in the network map, each column is a group, the keyword node size indicates the frequency, and the connection between keywords indicates the strength of the association. **(B)** Social frailty keyword clustering map, with a total of 179 nodes and 665 connections. Nodes with high homogeneity are clustered together, and different colors represent different clusters. **(C)** Top7 keywords with the strongest citation bursts, each blue line corresponds to a year, and the red line indicates that the keyword is frequently cited during that time period.

High-frequency keywords can reflect research hotspots in related fields, By tracking changes in keyword frequency over time, researchers can also observe how research interests and topics have evolved and adapted in response to relevant issues and developments in the field. The main metrics used in key co-occurrence analysis are frequency and centrality. Frequency measures how often a particular keyword appears in a dataset, indicating its relative importance in the field of study. Centrality, on the other hand, reflects the importance of nodes in a co-occurrence network. Nodes with high centrality have more connections with other nodes, indicating that they are important hubs in a network. They are often considered key drivers of research trends and hotspots in the field. The top five high-frequency keywords in Figure 7A are “social vulnerability” (101), “health” (96), “frailty” (79), “mortality” (74), and “older adult” (60). Keywords with high intermediation (centrality ≥0.1) are “health” (0.16), “risk” (0.14), “disability” (0.12), and “quality of life” (0.12), indicating that these are the main research focus areas in the field of social frailty. Figure 7B analyzes the distribution of high-frequency keywords over time. The closer the square color block color is to yellow, the higher the keyword popularity. In 2021–2022, the keyword color is mainly yellow, indicating that the popularity of social frailty research has continued to increase in the past 2 years. Figure 8A displays the keyword co-occurrence network plotted through VOSviewer. The timeline shows the average year, arranged in each column as a cluster, with the node size reflecting the importance of the keyword, while the darker the node color, the closer it is to 2020. This approach helps to better visualize the evolving research trends and hotspots in the field of social frailty.

The clustering process involves dividing the analyzed objects into groups based on the degree of correlation and grouping with a high degree of similarity. In the context of keyword analysis, the keyword cluster graph is extracted using the log-likelihood ratio (LLR) algorithm to extract keywords and plot. The cluster module value *Q* is set to 0.4582 (*Q* > 0.3), and the average contour value of the cluster S is set to 0.7559 (*S* > 0.5). This indicates that the clustering process is effective and reasonable. The larger the *S* value, the stronger the homogeneity (Shibata et al., 2008). In Figure 8B, the size of a cluster label corresponds to the number of keywords included in the cluster. The smaller the number of cluster labels, the larger the cluster size. The largest cluster is represented by 0# physical frailty, followed by 1# nutritional status and 2# anxiety, which are the top three clusters. This indicates that physical frailty, nutritional status, and anxiety are the most prominent research topics in the field of social frailty and have strongly overlapping keywords. The clustering analysis helps to identify groups of interrelated keywords and provides a comprehensive understanding of the research themes in the field of social frailty.

### 3.7 Keywords with citation bursts

Keyword with citation bursts can be performed using CiteSpace. To perform this analysis, the “Keyword” module should be selected, and the time partition should be set to “1” year. The “Top50” threshold should also be selected to identify keywords that are most relevant to the field of research. Additionally, the mutation analysis “burst terms” operation should be conducted to identify the research frontier in this field. By predicting the research trend and research frontier of a certain area, mutation term analysis can provide insights into the direction and focus of research in that field. It is important to note that the strength of the mutant word corresponds to the extent of the research trend of the keywords in the field. Figure 8C provides a list of the key words related to social frailty. The analysis shows that “dementia” was the earliest keyword that emerged in 2008 and continued to be relevant up until 2011. The keyword “Covid-19” (6.6277) had the greatest emergence and was the most timely, reflecting its recent and significant impact on social frailty research. In addition, “alzheimers disease” and “population” were identified as two keywords that have had the longest emergence within this field of study. Figure 8C analyzes keywords with the strongest citation bursts. The earliest keyword to appear in 2008 was “dementia.” “Dementia” was the first keyword to appear in 2008. The cited outbreak of “Covid-19” (6.6277) is the strongest and time-sensitive, indicating that it has had a significant impact on social vulnerability research in the short term. “Alzheimer’s disease” and “population” are the two keywords that have emerged in the field for the longest consecutive time.

## 4 Discussion

This study is based on data taken from 415 articles on social vulnerability authored by 1943 scholars in 254 journals listed in the Web of Science database from 2003 to 2022. After conducting an analysis of the data using CiteSpace, VOSviewer, and Biblioshiny, it was found that the number of articles related to social debilitation has increased exponentially over the last two decades. In 2022, there were 26 times as many articles as there were in 2004, reflecting a remarkable increase in attention in this area of research.

National publication volume indicates that Brazil has been the leading country in terms of publications and intermediary centrality in the field of social frailty related to older adults. The United States, with the largest number of independent publications, ranks second in total publications and has considerable influence. The differences between Brazil and the United States in terms of healthcare eligibility may influence perceptions of social frailty. Brazil’s provision of universal healthcare through SUS may result in fewer barriers for the elderly in accessing basic healthcare services, thereby associating social frailty more closely with social support, living conditions, and economic security. Conversely, in the United States, the structure of healthcare insurance may lead to greater financial burdens for elderly people when accessing specific types of healthcare services, thus linking social frailty more closely to healthcare costs, insurance coverage, and individual financial status. Despite these differences, the concept of social frailty may encompass multiple dimensions, such as health status, social participation, and economic resources, in both countries, with the specific policies and social structures of each influencing the relative importance of these factors.

The author analysis results reveal that Andrew MK, from the School of Geriatrics at Dalhousie University in Canada, is the top author in terms of the number of publications and total citations. In 2008, Andrew MK published a paper titled “Social Vulnerability, Frailty and Mortality in Elderly People,” which introduced the concept of social frailty using the deficit accumulation method. This study was the first to explore the relationship between social frailty, frailty, and mortality. The findings demonstrated a moderate correlation between social frailty and frailty, and a strong association of both with elderly mortality. This paper has been highly cited and is considered a seminal work in this field. Among the top 10 cited references, Andrew MK has three articles, including “Social Vulnerability from a Social Ecology Perspective: A Cohort Study of Older Adults from the National Population Health Survey of Canada” and “Social Vulnerability Predicts Cognitive Decline in a Prospective Cohort of Older Canadians.” These citation records illustrate the significant influence and noteworthy contributions of Andrew MK and her team in advancing the understanding of social debilitation.

Another highly cited article in this field is “Frailty and Cognitive Impairment – A Review of the Evidence and Causal Mechanisms” published by Robertson DA in 2013. This paper provides a comprehensive summary of the evidence and main pathological mechanisms related to frailty and cognitive impairment. It has received the highest number of overall and annual average citations and has made a significant impact on the field. Analysis of the cooperative network of cited authors demonstrates that there exist mature research teams in the area of social frailty, with close collaboration among them.

Among the top 10 journals with the highest number of publications on social frailty in the older adults, Brazil, the United States, and the United Kingdom each publish two journals. This corresponds with the national publication statistics, highlighting the notable contributions made by these countries in this field. In addition, the most cited journal in this area was J Am Med Dir Assoc (408) with a JCR subject category of Geriatrics & Gerontology and a JCR division of Q1. The journal’s high impact factor of 7.802 for 2021 can be attributed to the publication of high-quality and highly-cited articles.

High-frequency keywords results demonstrate that “social vulnerability” has the highest frequency, which aligns with the research theme. “Health” follows as a high-frequency and highly mediating keyword, indicating that social frailty is closely linked to the health of older adults and is often associated with adverse health outcomes such as mortality (Ma et al., 2018), physical function, cognitive impairment (Tsutsumimoto et al., 2017), and depression. Additionally, the high-frequency keyword “frailty” and the largest cluster, 0# physical frailty, reveal a growing body of research demonstrating that social frailty is linked to physical frailty. Frail symptoms predict the development of social frailty (Nagai et al., 2020). ”Risk,” “disability,” and “quality of life” are highly mediating keywords, revealing that the investigation of risk factors for social frailty is a current research focus. In a cohort study that explored frailty in older adults in Tanzania, the results suggest that social frailty is not only associated with frailty but also with mortality and disability (Cooper et al., 2022). Further linking social frailty and disability in older adults, Miriam Cappelli’s systematic review illustrates that social frailty is often associated with functional decline in ADL/IADL in terms of basic social needs, social resources, social behaviors and activities, and general social resources (Cappelli et al., 2020). Social frailty increases the risk of disability in older adults (Makizako et al., 2015). Thus, timely and effective social interventions can prevent or delay functional decline and death.

Keywords clusters suggest that nutritional status and psychological aspects can impact the social frailty of the older adults. In a meta-analysis (Verlaan et al., 2017), researchers found that malnutrition and frailty were associated with community-dwelling older adults, with 68 percent of malnourished older adults experiencing frailty. Moreover, nutrition plays a crucial role in improving frailty in older adults (Hoogendijk et al., 2020). Current research confirms the longitudinal association between social frailty and nutrition and diet in older men (Huang et al., 2020). however, this association has not been confirmed in older women, presenting a potential area for future research. Understanding the psychological status of socially fragile older individuals is equally important. Previous studies have determined that psychological robustness is a decisive factor in social frailty (Sugie et al., 2022). Additionally, the prevalence of social debilitation is higher in older adults experiencing negative emotions such as loneliness (Hoogendijk et al., 2020), depression (Tsutsumimoto et al., 2018), or anxiety (Henry et al., 2023). Therefore, medical interventions seeking to alleviate the social weakness of older adults should include screening for depression and nutritional management in addition to providing social resources and increasing social activities.

Keywords strongest citation bursts analysis highlights that the link between social frailty and disease has received increased attention with a research emphasis on social frailty-related diseases such as “dementia” and “Alzheimer’s disease.” A five-year longitudinal study found that frailty significantly increases an individual’s risk of transitioning from cognitive impairment to Alzheimer’s disease (Trebbastoni et al., 2017). Moreover frail older adults are eight times more likely to develop cognitive impairment and dementia than healthy individuals (Kulmala et al., 2014). Additionally older adults who exhibit a high degree of frailty may have more pathological features of Alzheimer’s disease and be diagnosed with dementia (Wallace et al., 2019). Japanese scholars Tsutsumimoto Kota (Tsutsumimoto et al., 2019) found that the social vulnerability of the elderly is closely related to Alzheimer’s disease (AD) incidence. However the direct relationship between social frailty and Alzheimer’s disease cannot be inferred. Consequently the question of whether preventative social vulnerability interventions can reduce the risk of Alzheimer’s disease requires further confirmation from high-quality studies

Furthermore “Covid-19” is the keyword with the greatest outbreak intensity. The COVID-19 pandemic has significantly impacted the global community’s health and wellbeing

especially the elderly population who are most at risk and vulnerable to the virus (Drane et al., 2021). Data suggests that one-quarter of COVID-19-related deaths occur among individuals aged 70–80 years and two-thirds of deaths occur among those over 80 years old (Calderón-Larrañaga et al., 2020). Since 2021 scholars worldwide have found that the social vulnerability of older populations has increased during the COVID-19 pandemic (Choi and Ko, 2022). Specifically

socially debilitated populations experience the health and economic consequences of COVID-19 leading to the continued accumulation of risks (Calderón-Larrañaga et al., 2020). Currently

global health responses have helped address the situation with risks associated with the frail elderly population gradually decreasing and the quality of life improving. The three words “population” “disability” and “united states” once again prove the analysis results of previous national cooperation papers and research hotspots. The keywords with the strongest citation bursts “population,” “disability,” and “United States “further validate the previous national cooperation papers’ analysis results and research hotspots

## 5 Strengths and limitations

This study utilized the Web of Science core database and leveraged three visualization software tools to provide a comprehensive analysis of the literature on social debilitation from various perspectives. However, there are certain limitations to our approach. Firstly, research on social weakness is still in its early stages, and our study only represents the current state of research. Secondly, our search was limited to a single database, which may have excluded potentially valuable information, and our results were restricted by search time and language (only English-language literature was included). In light of these constraints, future research should aim to conduct more inclusive systematic reviews of this area, incorporating a wider range of databases and addressing language limitations, to provide a more comprehensive exploration of the field of social debilitation.

## 6 Conclusion

This scientometric study explores social frailty in the field of older adults research, uncovering research hotspots and trends in the past 20 years. We identified the most influential countries, authors, and journals in the field, as well as the interrelationships between basic scientific knowledge and keyword research hotspots. Results demonstrate that the impact of social frailty on older adults’ health, such as the relationship between social frailty and frailty, mortality, disability, and the psychological and nutritional status of the socially frail older adults, are research hotspots. Additionally, the link between social frailty and disease is the current research trend and future research direction. We hope that this study will provide researchers with a better understanding of general trends in the field and potential research directions and partners for future research pursuits.

## Data availability statement

The original contributions presented in the study are included in the article/supplementary material, further inquiries can be directed to the corresponding author/s.

## Author contributions

HW: Conceptualization, Data curation, Funding acquisition, Investigation, Methodology, Project administration, Resources, Software, Validation, Visualization, Writing – original draft, Writing – review & editing. XC: Data curation, Formal analysis, Funding acquisition, Software, Supervision, Visualization, Writing – original draft, Writing – review & editing. MZ: Data curation, Investigation, Methodology, Software, Visualization, Writing – review & editing. YW: Data curation, Investigation, Methodology, Software, Visualization, Writing – review & editing. LL: Conceptualization, Data curation, Funding acquisition, Methodology, Project administration, Resources, Supervision, Writing – original draft, Writing – review & editing.
